# A transitional fossil mite (Astigmata: Levantoglyphidae fam. n.) from the early Cretaceous suggests gradual evolution of phoresy-related metamorphosis

**DOI:** 10.1038/s41598-021-94367-2

**Published:** 2021-07-23

**Authors:** Pavel B. Klimov, Dmitry D. Vorontsov, Dany Azar, Ekaterina A. Sidorchuk, Henk R. Braig, Alexander A. Khaustov, Andrey V. Tolstikov

**Affiliations:** 1 X-BIO Institute, Tyumen State University, Tyumen, 625003 Russia; 2 School of Natural Sciences, Bangor University, Bangor, LL57 2 UW UK; 3 Neurobiology of Development Laboratory, Institute of Developmental Biology of the Russian Academy of Sciences, Moscow, 119991 Russia; 4 Department of Earth and Life Sciences, Lebanese University, PO Box 26110217, Fanar, Lebanon; 5 Arthropoda Laboratory, Borissiak Paleontological Institute of the Russian Academy of Sciences, Moscow, 117997 Russia

**Keywords:** Entomology, Palaeontology

## Abstract

Metamorphosis is a key innovation allowing the same species to inhabit different environments and accomplish different functions, leading to evolutionary success in many animal groups. Astigmata is a megadiverse lineage of mites that expanded into a great number of habitats via associations with invertebrate and vertebrate hosts (human associates include stored food mites, house dust mites, and scabies). The evolutionary success of Astigmata is linked to phoresy-related metamorphosis, namely the origin of the heteromorphic deutonymph, which is highly specialized for phoresy (dispersal on hosts). The origin of this instar is enigmatic since it is morphologically divergent and no intermediate forms are known. Here we describe the heteromorphic deutonymph of *Levantoglyphus sidorchukae* n. gen. and sp. (Levantoglyphidae fam. n.) from early Cretaceous amber of Lebanon (129 Ma), which displays a transitional morphology. It is similar to extant phoretic deutonymphs in its modifications for phoresy but has the masticatory system and other parts of the gnathosoma well-developed. These aspects point to a gradual evolution of the astigmatid heteromorphic morphology and metamorphosis. The presence of well-developed presumably host-seeking sensory elements on the gnathosoma suggests that the deutonymph was not feeding either during phoretic or pre- or postphoretic periods.

## Introduction

The evolution of metamorphosis is thought to have generated an incredible diversity of organisms, allowing them to exploit different habitats and perform different functions at different life stages^1–5^. For example, the larval stage has a dispersal function in many marine organisms or it is specialized to exploit different food sources in holometabolous insects, while the adults’ main function is to find a mate and carry out sexual reproduction^4,6,7^. Metamorphosis permitted a number of subsequent evolutionary innovations, such as endoparasitism in insects, where the larva is typically parasitic, and adults reproduce and search for appropriate hosts^1^. The presence of disparate phenotypes in the life cycle of a single organism and, therefore, drastic metamorphosis occurring between them, presents an evolutionary enigma. The general consensus seems to be that complex metamorphosis is the outcome of natural selection acting on the phenotypes of both early and late life-history stages^8–11^. ‘Catastrophic’ or ‘drastic’ metamorphosis is generally assumed to be an outcome of gradual evolution acting differently on early and later developmental stages (so both acquire different evolutionary novelties) and reducing the duration of the intermediate phase^12–14^. Rapid punctuated evolution generating drastic metamorphosis may also occur through alternation of developmental pathways, as exemplified by non-feeding dispersal larvae of sea urchins^15,16^.

We use paleontological evidence to evaluate whether gradual or punctuated evolution was the main process that produced the complex life-cycle in astigmatid mites. These mites have two disparate phenotypes accomplishing different functions: (1) dispersing heteromorph (deutonymph that lost oral feeding and disperses via phoresy on hosts) and (2) feeding/reproductive homeomorphs (larva, protonymph, tritonymph, adults).

The Astigmata is a megadiverse lineage of microscopic arthropods comprising 77 families, 1128 genera, and 6150 species^17^, which represents only 3.5–6.8% of their estimated diversity at the species level^18^. This lineage originated within oribatid mites^19–21^, but unlike their ancestors, which are mostly restricted to soil, Astigmata shifted to a wide array of patchy and/or ephemeral habitats, particularly through associations with invertebrate and vertebrate hosts^22–25^. In human associations, the best known astigmatid mites are the economically important stored product mites (e.g., Acaridae, Glycyphagidae) and the medically important house dust mites (Pyroglyphidae) and scabies (*Sarcoptes*)^26^. Most habitats occupied by free-living astigmatid mites are patchy and ephemeral, which require specific adaptations to exploit these habitats: the ability to disperse (to quickly leave a depleted food source and arrive at a new one) and short life cycles, i.e., 1–4 weeks, to be able to complete development in short-lasting habitats. Typical ephemeral habitats harboring astigmatid mites are sporocarps of fungi, tree sap flows, dung, carrion, vertebrate and invertebrate nests, phytotelmata, decaying wood, and other organic matter^27^. In contrast, most oribatid mites live in a stable and continuous habitat (soil), which eliminates the need for long-range dispersal; their life cycles usually last from several months to well over a year (reviewed^28^).

**Figure 1:**
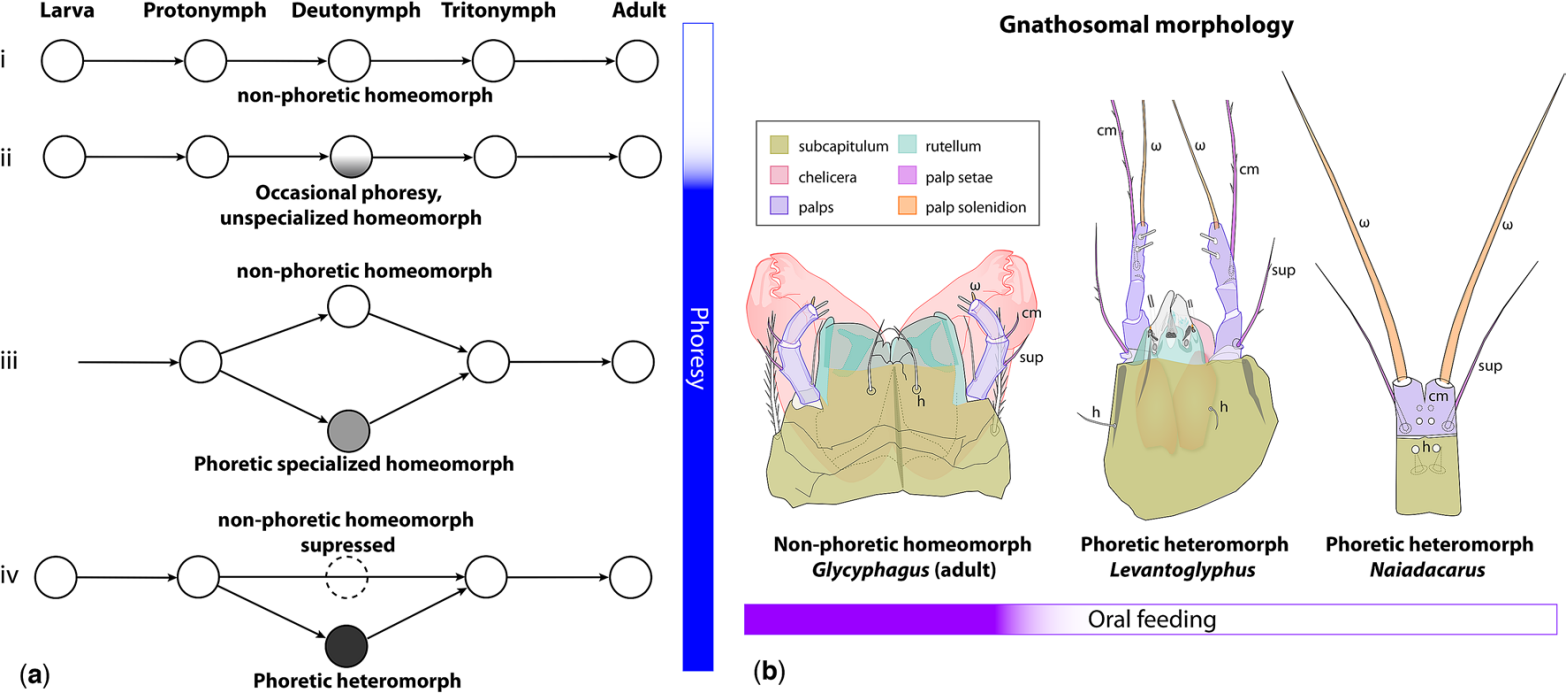
Hypothetical evolutionary series leading to the origin of the life-cycle with the heteromorphic deutonymph of Astigmata (**a**); (i) ancestral life cycle (Oribatida), protonymphal molt is unimodal, the deutonymph is unmodified and non-phoretic (homeomorphic); (ii) the protonymphal molt is unimodal, the deutonymph can be phoretic, but morphologically is unmodified (homeomorphic); (iii) the protonymphal molt is bimodal, producing either the non-phoretic unspecialized deutonymph (homeomorphic) or phoretic deutonymph with minor morphological adaptations for phoresy (specialized homeomorph); (iv) the protonymphal molt is bimodal, producing either the tritonymph (homeomorphic) or phoretic heteromorphic deutonymph; non-phoretic unspecialized deutonymph is suppressed; origin of phoresy is shown by the blue color on the vertical bar. Morphological diversity of the astigmatid mite gnathosoma, ventral view (**b**). Homologous gnathosomal parts are color-coded to emphasize sensory and masticatory elements: *Glycyphagus destructor* (Glycyphagidae), adult; *Levantoglyphus sidorchukae* n. gen. and sp. (Levantoglyphidae), phoretic heteromorphic deutonymph; *Naiadacarus arboricola* (Acaridae), phoretic heteromorphic deutonymph; presence/absence of oral feeding is shown by the violet color on the horizontal bar. Adult (male or female), *DN* deutonymph, *L* larva, *PN* protonymph, *TN* tritonymph.

One key innovation that enabled astigmatid mites to explore new habitats is the phoresy-related metamorphosis. In ancestral Astigmata, one developmental stage (the deutonymph), appearing in the middle of the life cycle (Fig. 1a), became highly specialized for dispersal through phoresy^24,28^. Phoresy is broadly defined as using a host as transport^29^, thus contrasting with parasitism where the dependent organism primarily derives nutrients from the host rather than dispersing. Oral feeding usually does not occur during phoresy in acariform mites, although obligate non-oral "retro-feeding" (i.e., via the anus or genital papillae) has been reported for a few astigmatid heteromorphic deutonymphs associated with insects and vertebrates^30,31^. These deutonymphs, therefore, are both phoretic and parasitic.

In all extant Astigmata, the dispersal stage (heteromorphic deutonymph, formerly known as hypopus) is drastically different from the remaining developmental stages. The heteromorphic deutonymph is well sclerotized (so it is more resistant to desiccation), the mouthparts are extremely reduced or absent, chelicerae are always lacking (Fig. 1b), the foregut is solid and non-functional (so no oral feeding is possible), the posteroventral body typically bears an attachment organ—a cluster of regularly arranged conoids and suckers that use adhesive forces and negative pressure for attachment, respectively. The origin of the heteromorphic deutonymph is a mystery. Extant taxa lack any intermediates that would give a clue about their early morphological evolution, so it may appear that the unique morphology of the heteromorphic deutonymph originated via a single punctuated change, leading to dramatic evolutionary modifications associated with phoresy and the loss of oral feeding. As R. Norton^28^ has put it in relation to a broader question on the origin of the astigmatid mite life history: "*If gradual physiological improvements were the key to the original success of the Astigmata, we might expect intermediate forms of life history in early-derivative groups, but there is no evidence of this*".

Here we describe exceptionally well preserved heteromorphic deutonymphs of astigmatid mites, *Levantoglyphus sidorchukae* n. gen. and sp., from early Cretaceous amber of Lebanon (ca. 129 Ma), displaying transitional morphology to extant taxa as compared to outgroups. Taking into account this fossil, we discuss the origin of the life-cycle of Astigmata, evidence for gradual evolution of the astigmatid heteromorphic deutonymph, and whether it could feed using its well-developed mouthparts.

## Results

### Systematic paleontology

Superorder **Acariformes** Zachvatkin, 1952

Order **Sarcoptiformes** Reuter, 1909

Suborder **Oribatida** van der Hammen, 1968

Hyporder **Astigmata** Canestrini, 1891

**Figure 2:**
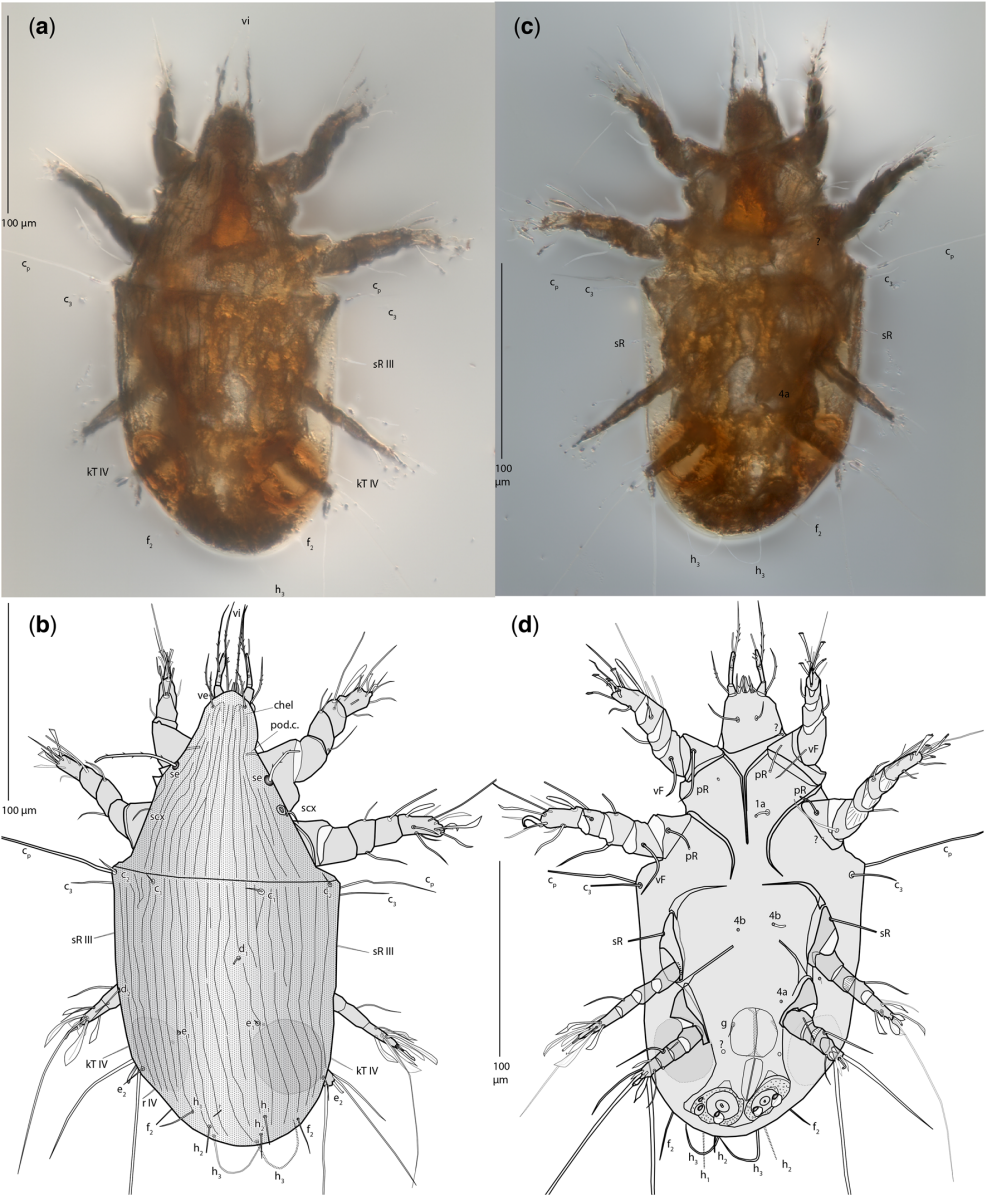
*Levantoglyphus sidorchukae* n. gen. and sp., phoretic heteromorphic deutonymph (holotype 1213A), dorsal view (**a**,**b**) and ventral view (**c**,**d**), line drawing (**b**,**d**) and photograph (**a**,**c**). Not all setae are well visible on photographs due to their low contrast; only well visible are labelled.

**Figure 3:**
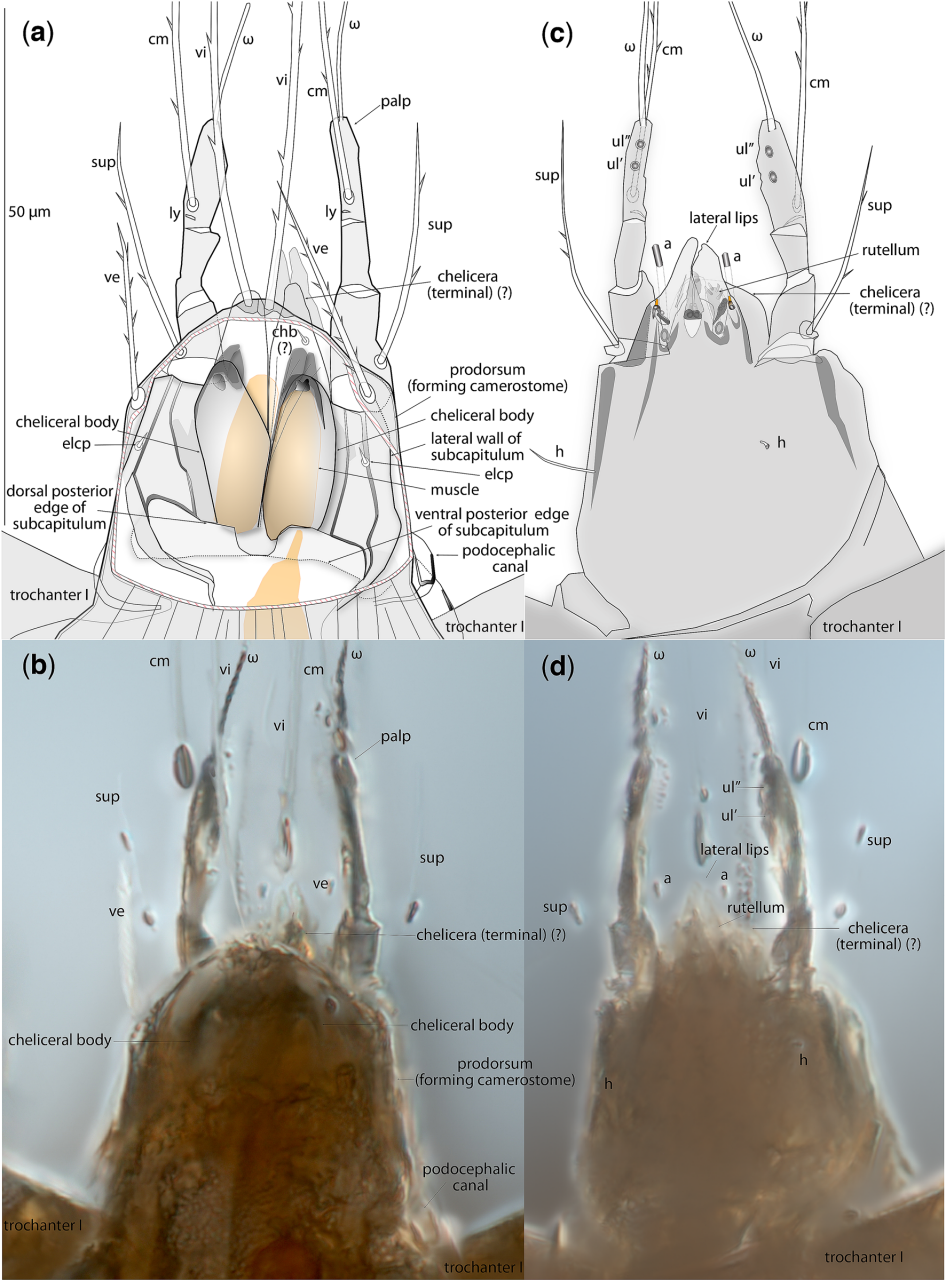
*Levantoglyphus sidorchukae* n. gen. and sp., phoretic heteromorphic deutonymph (holotype 1213A), gnathosoma, dorsal view (**a**,**b**), dorsal portion of camerostome removed to show internal structures (**a**), ventral view (**c**,**d**), line drawing (**a**,**c**) and photograph (**b**,**d**). Not all structures are well visible on photographs due to their low contrast.

**Figure 4:**
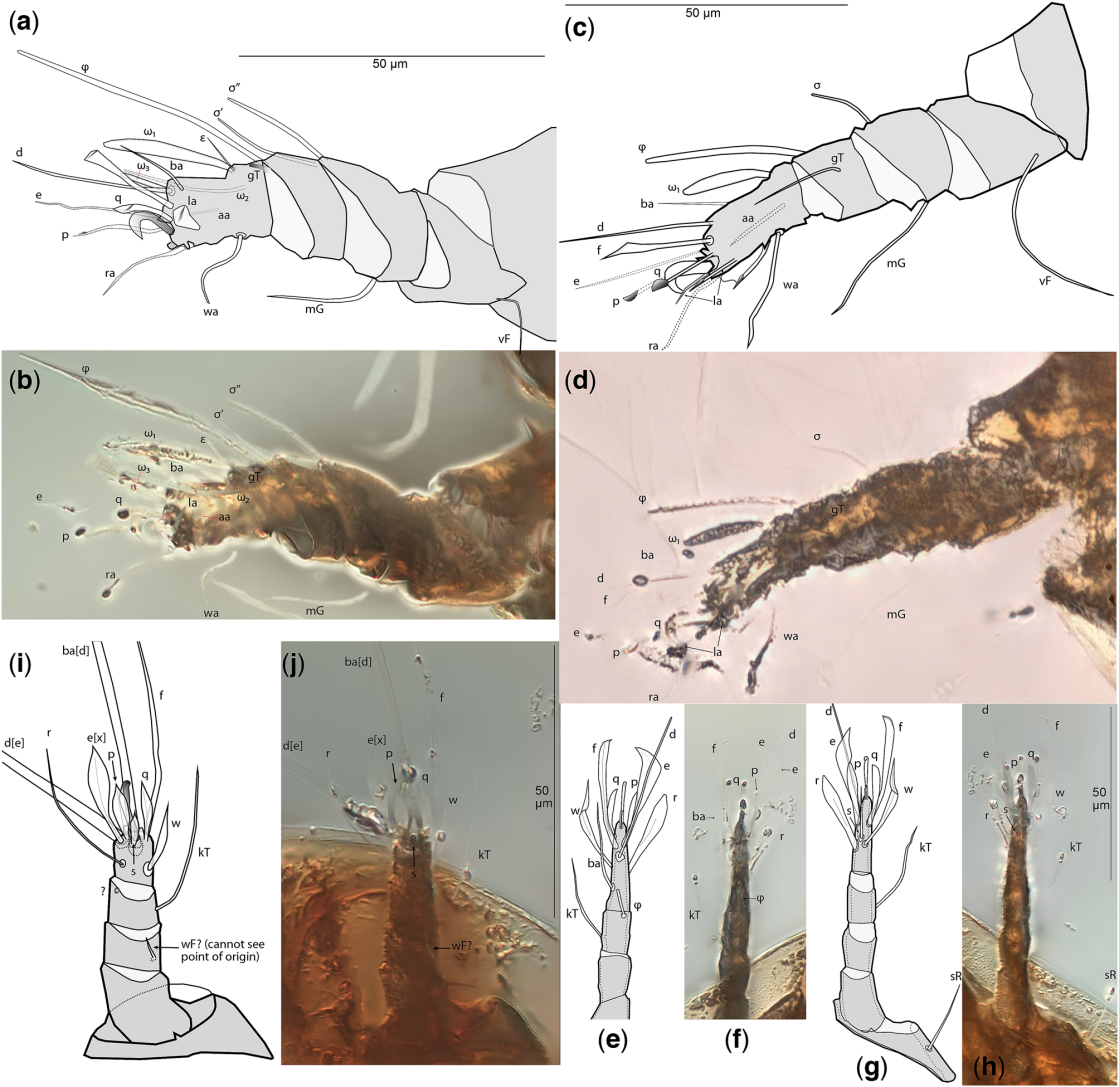
*Levantoglyphus sidorchukae* n. gen. and sp., phoretic heteromorphic deutonymph (holotype 1213A), legs I–IV (**a**–**j**): right leg I (**a**,**b**), ventrolateral view (most of anterior side shown); right leg II (**c**,**d**), ventrolateral view (most of anterior side shown); right leg III (**e**–**h**) dorsal view (**e**,**f**), ventral view (**g**,**h**), line art; right leg IV (**i**,**j**). Line drawing (**a**,**c**,**i**,**e**,**g**), photograph (**b**,**d**,**f**,**h**,**j**). Not all setae are well visible on photographs due to their low contrast.

Family **Levantoglyphidae** n. fam. (Figs. 2, 3, 4, Supplementary information 1)

#### Diagnostic description (heteromorphic deutonymph)

Unique apomorphies: Palpal solenidion ω and palpal setae *cm* and *sup* elongated and barbed; genual setae III–IV relatively elongate. Plesiomorphies indicating that Levantoglyphidae is a stem group of Astigmata: palps 4-segmented, with tarsus, fused genu + tibia, femur, and small trochanter; gnathosoma, cheliceral bodies, labrum, and rutella well developed; podocephalic canal and supracoxal opening developed; anal opening large, nearly as long as attachment organ; solenidion ω_2_ on tarsus I long, filiform; tarsus II with seta *aa* present; tarsi III–IV with 9 setae (setae *ba* III–IV present). These 11 unambiguous plesiomorphic character states of Levantoglyphidae also imply the presence of the corresponding 11 unambiguous synapomorphies of the crown group Astigmata. Other diagnostic characters (polarities unclear): external vertical setae *ve* present; dorsal idiosoma longitudinally striated; genu I with two well-developed dorsal solenidia (σ′ and σ″'); tarsal setae *aa* and *ba* I present on tarsus I; tarsal setae *e* I–II without terminal “saucer”; tarsus I with solenidion ω_3_ apical, widely separated from basal solenidia ω_1_ and ω_2_; legs IV shorter than legs III; tarsal apical setae *d* III and IV elongated; tarsal apical setae *ba* and *f* IV elongated. A detailed description is available in Supplementary information 1.

Genus ***Levantoglyphus*** n. gen. (Figs. 2, 3, 4, Supplementary information 1)

Type species: *Levantoglyphus sidorchukae* **n. sp.**

#### Etymology

Levantoglyphus is a compound noun, gender masculine, formed from Levant (Lat., a geographical area referring to the countries bordering the eastern Mediterranean Sea, including Lebanon) and glyphus (from Greek γλῠ́φω, to carve, cut out with a knife, engrave), which is used to form compound genus-group names in Astigmata.

#### Diagnosis

Because *Levantoglyphus* is a single genus in the family Levantoglyphidae*,* we give select diagnostic, genus-level characters of other families of Astigmata in Supplementary information 1. Most important character states are internal vertical setae (*vi*) more than twice as long as external vertical setae (*ve*); their bases separated (not contiguous); tarsus IV with 2 apical setae (*d* and *ba*) longer than the length of leg IV; 1 apical seta (*f*) as long as the length of genu-tarsus IV; and 1 seta (*r*) nearly as long as the length of genu-tarsus IV.

***Levantoglyphus sidorchukae n. sp.*** (Figs. 2, 3, 4, Supplementary information 1)

#### Etymology

The new species is named after the late Ekaterina (Katya) Sidorchuk who made major contribution in palaeoacarology. She initiated this project and accomplished critical tasks during its early stages (polishing the amber piece and partial imaging).

#### Material

Holotype: Heteromorphic deutonymph, specimen number 1213A (Azar collection), dorsoventral orientation, LEBANON: Mouhafazet Jabal Loubnan [Governorate of Mount Lebanon], Caza Baabda [Baabda District], Hammana—Mdeyrij outcrop, date and collector unknown (deposited in Natural History Museum of the Lebanese University, Faculty of Sciences II, Fanar, Lebanon). The piece is mounted in epoxy resin between two coverslips. Geological map of this locality is given in Azar et al.^32^. The age of the outcrop is Lower Barremian, ca. 129 My^33,34^.

Paratype: Heteromorphic deutonymph, specimen number 1213B (Azar collection), lateral orientation, same amber piece as holotype.

#### Description (heteromorphic deutonymph)

This species belongs to a monotypic genus having remote similarities with modern astigmatid mites. Because there is no other species to compare to (or even make an informed guess based on other astigmatid mites), we consider that the family and generic descriptions above are equivalent to the species description. Basic measurements are as follows: length of idiosoma 232(160), width 116 μm; propodosoma 96(65) μm long; hysterosoma 136(98) μm long (measurements are given for the holotype, and, in parentheses, for the paratype). Other measurements and species-level diagnostic character states can be derived from photographs (Figs. 2, 3, 4, Supplementary information 1).

#### Remarks

Similarly to heteromorphic deutonymphs of crown group Astigmata, the fossil mite is well sclerotized, has the attachment organ and many foliate tarsal setae, and long apical setae on tarsi III–IV. Based on comparison with living mites, these character states collectively suggest that *Levantoglyphus* was phoretic on arthropods. But unlike all extant Astigmata, the gnathosoma, including the rutella and chelicerae (main structures participating in shredding food particles during feeding), are well developed in the fossil mite. These and some other character states, such as presence of setae *ba* on tarsi III–IV and setae *aa* on tarsi II, suggest that these mites have no close modern relatives, and therefore belong to the stem group Astigmata. We also note that the astigmatid genus *Schizoglyphus* (Schizoglyphidae) also has a number of plesiomorphic character states (3-segmented palps, 3 pairs of genital papillae)^24^ suggesting that it is sister to all other extant Astigmata, but in comparison with *Levantoglyphus* its gnathosoma is reduced (similarly to other Astigmata) and tarsal setae *aa* II and *ba* III–IV are absent. *Levantoglyphus sidorchukae* differs from an undescribed astigmatid deutonymph from the Cretaceous termite *Lebanotermes veltzae*^35^ by the elongated propdosoma (much shorter in the termite mite). There are only five published records of fossil astigmatid heteromorphic deutonymphs: (1) An undetermined astigmatid mite phoretic on *Lebanotermes veltzae* Engel, Azar and Nel, 2011 from Lebanese amber, 129 Mya^35,36^; (2) Acaridae or Histiostomatidae, from the spider *Dasumiana emicans*, Baltic amber, Eocene, 44–49 Ma^37^; (3) *Histiostoma ovalis-*species group (Histiostomatidae) from a bark beetle of the genus *Phloeosinus*, Baltic amber, Eocene, 44–49 Ma^38^; (4) *Winterschmidtia* or *Parawinterschmidtia* (original genus *Amphicalvolia*), family Winterschmidtiidae, Mexican Chiapas amber, Miocene, 23.03–15.97 Ma^39^; (5) An undetermined astigmatid mite from an ambrosia beetle, subfamily Platypodinae, Dominican amber, Miocene, 20.4–13.7 Ma^40^. The oldest known fossil records of non-phoretic stages of Astigmata are also from the Eocene Baltic amber^41,42^. *Levantoglyphus sidorchukae* represents the second record of Astigmata and its phoretic behavior with the minimum age of 129 Ma.

## Discussion

Metamorphosis is a key innovation allowing the same species to inhabit different environments and accomplish different functions, leading to evolutionary success in many animals^1–5^. Although much progress has been made to understand the origin of metamorphosis, producing complex life-cycles with phenotypically disparate phases, this question still remains enigmatic and oftentimes controversial^8–11,15,16^. Paleontological evidence is especially important here because it can distinguish whether evolution of metamorphosis was a continuous accumulation of changes over a long period of time (gradual evolution)^12–14^ or it occurred rapidly without intermediate steps (punctuated evolution), for example, as a result of alternation of developmental pathways^15,16^. Here we present a detailed comparative morphological comparison of an exceptionally well-preserved Cretaceous fossil mite, *Levantoglyphus sidorchukae*. This is a heteromorphic deutonymph capable of phoresy on arthropod hosts and displaying a transitional morphology, particularly in having its mouthparts well-developed (unlike extant deutonymphal Astigmata having vestigial mouthparts). Here we integrate this critical fossil with our knowledge about phoretic morphology and biology of extant mites, and try to distinguish between the two scenarios in the evolution of the complex life-cycle of Astigmata.

In acariform mites, only a single instar is specialized for phoretic dispersal, deutonymph or adult (female only or both sexes). The degree of specialization of the phoretic stage can be classified into unspecialized homeomorphs, specialized homeomorphs, and heteromorphs having, respectively, no, minor, or drastic differences as compared to non-phoretics^25^. Phoretic individuals typically do not feed using mouthparts; phoretic adults do not mate or deposit eggs while on host.

Unspecialized homeomorphs disperse by attaching to the host using preexisting structures normally adapted for other purposes, such us the claws and/or membranous pretarsal ambulacra. The examples are: males and females of *Aeroglyphus peregrinans* (Astigmata) phoretic on carpenter bees^43^; numerous genera of adult oribatids (males and females), *Licnocepheus*, *Scheloribates*, *Euscheloribates*, *Oppia*, *Paraleius*, phoretic on various insects^44,45^; and numerous Heterostigmata where inseminated females are usually phoretic, while males are short-lived and non-feeding (e.g., *Trochometridium*, *Pediculaster*, *Petalomium*, *Scutacarus*, *Heterotarsonemus*, *Iponemus*, *Pseudotarsonemoides*, *Nasutitarsonemus*, *Tarsanonychus*, *Tarsonemella*, *Pseudacarapis*, *Suctarsonemus*, *Pseudotarsonemus*, and some species-groups of *Tarsonemus*)^46–50^. Similarly to Heterostigmata, phoresy is common for females of Cheyletidae^51^. The oribatid mite genus *Mesoplophora* presents a unique case where its phoretic adaptations are based on a complex morphology evolved as defense against small predators (i.e., ptychoidy). The propodosoma of these mites can fold over and cover the soft cuticle and appendages of the mite, which are most vulnerable to attacks. At the same time, the folded propodosoma can serve as part of a clasping mechanism that is used by the mite to cling to the setae of insect hosts during phoresy^44^.

Specialized homeomorphs have relatively minor morphological adaptations for phoresy as compared to non-phoretic individuals. There is only one instance in oribatids—some species of the genus *Mesoplophora* have phoresy-specific morphological modifications: each genital plate of the adult mite has a tooth-like tubercle. When the mite propodosoma is closed on a host seta, these tubercles act as a locking mechanism, preventing the seta from slipping out^44^. In contrast to oribatids, specialized homeomorphs are common in Heterostigmata, where this stage is called phoretomorph. They are female-only and they are optional (facultative) in the life-cycle and can be produced alongside normal, non-phoretic females. Phoretomorphs occur in several heterostigmatid genera, e.g., *Pyemotes*, *Pediculaster*, *Archidispus, Lamnacarus, Scutacarus*^48,52–55^. These females display conspicuous modifications for phoresy: the first pair of legs and their claws are greatly enlarged and are used to grasp the host setae or its soft intersegmental skin^54^. Some phoretomorphs (*Archidispus*) also show thickening of several ventral setae^54,56^, a condition which is reminiscent of conoidal setae of astigmatid heteromorphic deutonymphs, such as coxal setae *1a*, *3a*, and *4b* of *Chaetodactylus krombeini*. Furthermore, in many Heterostigmata, especially in Tarsonemidae and Scutacaridae, legs IV are modified, they are shorter than legs III and have several very long setae (as in the fossil mite and many modern phoretic Astigmata). These legs are used for standing in an upright position as part of host-seeking behavior, with the long setae of legs IV providing further support and backing when in bipedal stance. While in the upright position, mites make questing movements with their first pair of leg (furnished with many sensory solenidia and sensilla) probing the environment for various clues emitted by the host. During normal locomotion, anterior legs are not greatly exposed and legs IV and their long setae are trailed. In a few species of the genera *Imaripes* and *Lophodispus*, the long setae of legs IV are used for jumping, either to lodge on the host or evade predators^56,57^. Jumping could be an effective adaptation for mites targeting quickly moving or flying hosts, e.g., flies.

Heteromorphs occur only in Astigmata. As detailed in “Introduction” section, the heteromorphic deutonymph is a facultative, immature instar typically adapted for phoresy. In all extant Astigmata that retain this instar, heteromorphic deutonymphs are drastically different from the remaining developmental instars (see “Introduction”). In several independent lineages, hereromorphic deutonymphs are bimorphic, with one type being phoretic and mobile and another one is immobile, having extremely reductive, nearly featureless, sac-like morphology (immobile heteromorphic deutonymph). The latter deutonymph is a quiescent instar that can withstand environmental stresses for prolonged periods of time. For example, it can wait in an abandoned nest cavity until it is re-used by a new host^58^. When deutonymphal dimorphism is present, the preceding ontogenetic stage, the protonymph, can molt into either of these two types of deutonymphs or the tritonymph. So the outcome of the protonymphal molt is trimodal. When only the phoretic deutonymph is present (most typical case), the outcome of this molt is bimodal (deutonymphs or tritonymph) (Fig. 1b). Exceptions from these rules are: *Tensiostoma* (Histiostomatidae) with deutonymphal dimorphism but the molt from protonymph to tritonymph does not occur (bimodal)^59^; many astigmatid mites do not form deutonymphs (e.g., *Tyrophagus*, Psoroptidia), the protonymph can only molt to tritonymph (unimodal)^24^. Similarly to Heterostigmata (see above), astigmatid heteromorphic deutonymphs can adopt a questing posture, standing in a tilted position on legs III and IV and making questing movements with legs I and II e.g., in Histiostomatidae and Acaridae^60,61^. Deutonymphs of *Hormosianoetus laboratorium* can jump up to 5 cm using a downward push of legs III (oriented anteriorly), while their legs IV are oriented posteriorly^62^. Legs IV probably have no function in jumping or walking, and are dragged along behind while the mite is walking^62^.

The mouthparts and foregut of extant astigmatid heteromorphic deutonymph are reduced and non-functional, making oral feeding impossible. Oral feeding can only start when the deutonymph molts to the next stage, the tritonymph. The tritonymphal stage is homeomorphic, has normally developed mouthparts and digestive system, and can feed. Unlike all modern astigmatid phoretic deutonymphs, *Levantoglyphus sidorchukae* has a developed gnathosoma. The presence of chelicerae (with internal cheliceral muscles) and rutella, which are the main structures used to shred food particles, may suggest that oral feeding could occur at this stage. However, it is highly unlikely based on the known biology of extant mites—the lack of oral feeding during phoresy seem to be the general rule for acariform as well as parasitiform mites. For example, phoretomorph females of Heterostigmata, phoretic adult oribatid mites, and many parasitiform mites have functional mouthparts but they do not feed during phoresy^18,24^. When these mites disembark from the host in a suitable habitat, they start feeding and continue their life cycles. There a few recorded cases, all involving homeomorphs, of mites that can feed during phoresy: the parasitform mite *Poecilochirus carabi*^63^ and probably astigmatid mites of the *Sennertia vaga* species group^64^. Because no known specialized homeomorphs or heteromorphs of extant mites can feed during phoresy, the likelihood of feeding for *Levantoglyphus* heteromorphic deutonymphs seems extremely low. However, could *Levantoglyphus* deutonymphs feed in the pre- and postphoretic periods? This is known for Heterostigmata, and *Levantoglyphus* deutonymphs do have a feeding-ready gnathosoma, with both chelicerae and rutella well-developed. We believe that feeding in this situation is also not likely. There are several modifications suggesting that the gnathosoma of the fossil mite was primarily sensorial, therefore, replacing its principal function of processing and manipulating food. A comparison of the general astigmatid adult gnathosoma (feeding instar) and the typical gnathosoma of the phoretic deutonymph (non-feeding), suggests that, while nearly all elements of the typical "feeding" gnathosoma are present (i.e., color-coded elements of *Glycyphagus* and *Levantoglyphus* in Fig. 1b), several sensorial features are enlarged and developed in a way similar to extant phoretic deutonymphs (i.e., *Levantoglyphus* vs *Naiadacarus*). The above sensorial elements include extremely elongated palpal solenidia (ω) and setae *cm* and *sup* (Fig. 1b), with solenidia being olfactory chemoreceptors and setae being mechanoreceptors^65^. It seems, therefore, conceivable that the disproportionate development of these sensory elements occurred in response to the need of phoretic host seeking, not as part of sensing or testing food. Based on this argument, we believe that the deutonymphal gnathosoma of *Levantoglyphus* was sensorial, and the mite, therefore, was not feeding at the entire deutonymphal stage.

Heteromorphic deutonymphs of extant Astigmata lack any intermediates that could shed light about their early morphological evolution. The most conspicuous changes characterizing extant heteromorphic deutonymphs are dramatic transformations of the gnathosoma and digestive system—functional mouthparts and foregut are absent (see “Introduction”). Based on the mostly reductive nature of these modifications and the absence of known intermediate morphologies, it seems possible that the origin of the heteromorphic deutonymph is a result of a single, rapid punctuated change^28^. Interestingly, punctuated evolution has been shown to occur through alternation of developmental pathways, as was exemplified by non-feeding dispersal larvae of sea urchins that can return to their ancestral state of feeding larvae^15,16^. Taking into account the transitional gnathosomal morphology of *Levantoglyphus* and comparative aspects related to phoresy discussed above, we suggest that the ancestral evolution of Astigmata (1) started from the trinymphal ontogenesis typical for oribatid mites (Fig. 1a); then proceeded to (2) the deutonymph capable of occasional phoresy on arthropod hosts (unspecialized homeomorph); the outcome of the next step was (3) the evolution of deutonymphal dimorphism, with specialized phoretic homeomorphs and non-phoretic homeomorphs; (4) following by further evolution of the former to a heteromorphic deutonymphal stage and the suppression of the latter stage. States (2) (unspecialized homeomorphs) and (3) (specialized phoretic homeomorphs) are seen in many linages of modern Heterostigmata (see above). State (4) occurs in modern Astigmata. *Levantoglyphus sidorchukae* presents an intermediate step between states (3) and (4). The presence of many phoresy-related characters shared with modern Astigmata (attachment organ, foliate tarsal setae, long terminal setae on posterior legs probably assisting in questing behavior and/or jumping, modified legs IV) leaves almost no doubt that this mite was phoretic. However, it has a well-developed gnathosoma, which is strongly reduced in all modern astigmatid phoretic deutonymphs (Fig. 1b). This feature points that the evolution of the phoretic heteromorphic deutonymph, and by extension the entire astigmatid life-history (see “Introduction”), was gradual rather than punctuated.

## Methods

### Amber source and preparation

The amber piece was cut and polished following published protocols^66,67^ and using tools as described previously^68^. Briefly, this technique is based on polishing amber until very small pieces containing the specimens are obtained. The distance from the amber surface to the mite specimen does not exceed 100 μm, which allows use of the highest resolution oil immersion optics (100× microscope objective). As a result, the finest details, such as the leg and body setation can be observed. The polished piece was mounted in Buehler Epo-Thin epoxy resin between two coverslips. In the course of the microscopic study, one of the coverslips was inadvertently damaged, which led to the immersion oil getting inside the inclusion, making it more transparent. This opportunity was used to produce additional stacks of images, revealing fine structure of the gnathosoma.

### Imaging

Compound microscopes Nikon E-800 with water immersion optics (40× and 60×), Zeiss AxioImager A2 with dry (40×, 63×) and oil immersion optics (100×), Leica DM 2500 LED (40×, 100×). Brightfield, phase contrast, polarized and differential interference contrast and incident illumination were used. Stacks of images, comprising multiple focal planes, were obtained with digital cameras Nikon D-7000 on the Nikon microscope, Zeiss Axiocam 506 color, Hitachi KP-HD20A on the Zeiss microscope, and Leica DMC4500 on the Leica microscope. Stacks were corrected for colour, brightness and noise with Adobe Lightroom, then exported to JPEG or TIFF format. The resulting original stacks are available through Figshare (10.6084/m9.figshare.c.4657688), along with the technical details (magnification, camera, and illumination). Finished figures were produced from 20 to 200 layered (multifocal) images processed with Helicon Focus 6.8.0, using algorithms A (mostly) and B with subsequent manual addition of significant details from the individual focal planes. A similar focus stacking software, Zerene Stacker v 1.04, was also used with the PMax option. Drawings were prepared with Adobe Photoshop CS6 or Illustrator CC, using image stacks as a background^69^. In case of very large stacks, a series of multifocal images were assembled first (see above) and then 1–3 composite stacked images were used as a background to trace and produce vector line art by hand.

The fossil specimens were not flattened (as is the case of for specimens mounted with the conventional technique widely used for extant mites), and their setae were oriented nearly vertically to the surface (as in live specimens), making it difficult to observe these setae. However, in amber fossils, vertically oriented setae can sometimes be easily identified by tiny bubbles of air near their tips; but unfortunately, sometimes the points of origin of these setae could not be seen, even with phase contrast optics. These setae were drawn without their basal parts and, if possible, were identified based on their relative positions.

### Terminology

Idiosomal chaetotaxy follows Griffiths, et al.^70^; the terminology of coxisternal setae follows Norton^19^; for appendages, the chaetotaxy and solenidiotaxy follow Gradjean for palps^71^ and for legs^72^.

## Supplementary Information

Supplementary Information.
