# Supplementary material for: A transitional fossil mite (Astigmata: Levantoglyphidae fam. n.) from the early Cretaceous suggests gradual evolution of phoresy-related metamorphosis
Source: Sci Rep. 2021 Jul 23;11:15113. doi: 10.1038/s41598-021-94367-2 (PMC8302706; doi:10.1038/s41598-021-94367-2)
Supplement: Supplementary file 1 — Supplementary Information. [file 41598_2021_94367_MOESM1_ESM.docx]

# **A transitional fossil mite (Astigmata: Levantoglyphidae fam. n.) from the early Cretaceous suggests gradual evolution of phoresy-related metamorphosis**

Pavel B. Klimov^1,2*^, Dmitry D. Vorontsov^3^, Dany Azar^4^, Ekaterina A. Sidorchuk^1,5^, Henk R. Braig^2^, Alexander A. Khaustov^1^, Andrey V. Tolstikov^1^

^1^X-BIO Institute, Tyumen State University, Tyumen, 625003, Russia.

^2^School of Natural Sciences, Bangor University, Bangor, LL57 2 UW, UK.

^3^Neurobiology of Development Laboratory, Institute of Developmental Biology of the Russian Academy of Sciences, Moscow, 119991 Russia.

^4^Department of Earth and Life Sciences, Lebanese University, Fanar, PO box 26110217, Lebanon.

^5^Arthropoda Laboratory, Borissiak Paleontological Institute of the Russian Academy of Sciences, Moscow, 117997, Russia.

*email: pklimov@umich.edu

**Supplementary Description of *Levantoglyphus* *sidorchukae* gen. and sp. n. (Levantoglyphidae, fam.n.)**

Superorder **Acariformes** Zachvatkin, 1952

Order **Sarcoptiformes** Reuter, 1909

Suborder **Oribatida** van der Hammen, 1968

Hyporder **Astigmata** Canestrini, 1891

Family **†Levantoglyphidae** **Klimov and Sidorchuk, n. fam.** (Figs 3–4, Supplementary Figs S1-8)

*Diagnosis*. Unique apomorphies: Palpal solenidion ω and palpal setae *cm* and *sup* elongated and barbed; genual setae III-IV relatively elongate. Plesiomorphies indicating that Levantoglyphidae is a stem group of Astigmata: palps 4-segmented, with tarsus, fused genu+tibia, femur, and small trochanter; gnathosoma, cheliceral bodies, labrum, and rutella well developed; podocephalic canal and supracoxal opening developed; anal opening large, nearly as long as attachment organ, solenidion ω_2_ on tarsus I long, filiform; tarsus II with seta *aa* present; tarsi III-IV with 9 setae (setae *ba* III-IV present). Other diagnostic characters (polarities unclear): External vertical setae *ve* present; dorsal idiosoma longitudinally striated; genu I with two well-developed dorsal solenidia (σ' and σ''); tarsal setae *aa* and *ba* I present on tarsus I; tarsal setae *e* I-II without terminal "saucer"; tarsus I with solenidion ω_3_ apical, widely separated from basal solenidia ω_1_ and ω_2_; legs IV shorter than legs III; tarsal apical setae *d* III and IV elongated; tarsal apical setae *ba* and *f* IV elongated.

*Description (heteromorphic deutonymph).* Body. Gnathosoma well-developed, with chelicerae, rutella, and labrum. Palp with 4 freely articulating segments: tarsus, fused genu+tibia, femur, and small trochanter. Body ovate. Relatively small, not broadly tear-drop shaped. Prodorsal and hysterosomal dorsal sclerites not separated by soft cuticle at sejugal region, covering entire body dorsally; both sclerites longitudinally striated and finely punctate. Ocelli absent; pigmented eye-spots not observed. Propodosomal sclerite long, longer than half of hysterosomal length. Internal and external vertical setae (*vi* and *ve*) present, long, situated on anterior dorsal propodosoma (not on a protuberance or ventral tubercle between gnathosoma and overhanging propodosoma). External scapular setae (*se*) present, situated on lateral sides of propodosomal sclerite; internal supracoxal setae (*si*) not observed. Podocephalic canal, supracoxal setae (*scx*) and supracoxal gland openings present. Dorsal opisthosomal setae filiform, not bifurcate. Cupules not observed. Coxal fields II, III, IV open. Coxal apodemes II-IV end freely. Coxal fields III not narrowing medially, and not connected by a short apodeme. Sternum well developed, distinctly not reaching level of anterior apodemes III; posterior apodemes II not observed; hysterosomal ventral median apodeme not observed. Coxal setae *1a* and *3a* present; setae *4a* and *4b* not observed. Progenital valves situated ventrally, not surrounded by a sclerotized ring, with single pair of setae (g). Genital papillae not observed. Attachment organ well developed. Median suckers (*ad_1+2_*) well developed. Anterior suckers (*ad_3_*) not observed. Setae *ps_1_* and *ps_2_* in the form of conoids. Anal opening large, nearly as long as attachment organ.

Legs. Legs I-IV with all segments developed, none fused; Legs III-IV not flexed at trochanter-femur articulation and directed posterior laterally. Leg IV shorter than leg III, with three long apical setae (*d*, *ba*, *f*), of which *f* is shorter than the others (*d*, *ba*). Tarsi I-IV with empodial claws present, simple (not large and hooked), arising on tarsal apices and connected with relatively short, thin and symmetrical condylophores, ambulacra inconspicuous (claws are not borne on large membranous ambulacra). Tarsus I with solenidion ω_3_ apical, widely separated from basal solenidia ω_1_ and ω_2_. Solenidion ω_2_ on tarsus I long, filiform. Seta *ba* and *aa* present on tarsi I-II; tarsal setae *e* I-II without terminal "saucer". Tarsus II with one solenidion (ω_1_). Tarsi III-IV with 9 setae (8 typical astigmatid setae plus setae *ba* III-IV). Genu I with two well developed dorsal solenidia (σ' and σ"). Genu II with one dorsal solenidion (σ). Tibiae I-II with single anterior ventral seta (*gT* I-II present, *hT* I-II absent). Ventral setae of tibiae III-IV (*kT*) filiform, unmodified.

*Remarks*. This family is based on a single monotypic genus *Levantoglyphus* n. gen. found in Cretaceous amber of Lebanon (see below). Unobserved characters: internal scapular setae (*si*); cupules, posterior apodeme of coxal field II; dorsal genual setae *cG* I-II, ventral genual setae *nG* III; genual solenidion σ III, and tibial solenidion φ IV. Generally, the ventral region, including attachment organ, coxal fields, and coxal setae, is challenging for accurate observations due to folding as the result of preservation (e.g., the attachment organ region is folded inwards in the holotype specimen). The presence of dark muscles, other tissues, unrecognizable internal body particles, cracks in amber (significant around legs I-II), and air bubbles also complicate light microscopic observations.

Genus **†*Levantoglyphus* Klimov and Sidorchuk, n. gen.** (Figs 2–4, Supplementary Figs S1-8)

Type species: †*Levantoglyphus* *sidorchukae* Klimov and Sidorchuk, **n. sp.**

*Etymology*. Levantoglyphus a compound noun, gender masculine, formed from Levant (Lat., a geographical area referring to the countries bordering the eastern Mediterranean Sea, including Lebanon) and glyphus (from Greek γλῠ́φω, to carve, cut out with a knife, engrave), which is used to form compound genus-group names in Astigmata.

*Diagnosis*. Because *Levantoglyphus* is a single genus in the family Levantoglyphidae*,* we give select diagnostic, genus-level characters of other families of Astigmata in the Description section below.

*Description (heteromorphic deutonymph)*. Body. Dorsal body surface longitudinally striated and finely punctate. Pigmented eye-spots and distinct lenses absent from dorsal idiosoma. Internal scapular setae (*si*) not observed or absent; otherwise full complement of dorsal idiosomal setae present, setae elongate and filiform, setae *ve*, *vi*, and *se* barbed, other idiosomal setae smooth. Internal vertical setae (*vi*) more than twice as long as external vertical setae (*ve*); with bases separated (not contiguous). Supracoxal seta (*scx*) simple and filform (not bifurcate apically). Posterior dorsum without longitudinal apodemes. Opisthonotal gland openings situated dorsally near setae *e_1_*. Coxal fields not heavily sclerotized. Coxal apodemes I fused into a sternal apodeme (sternum) that ends far anterior to ends of coxal apodemes II. Anterior coxal apodemes II well developed, but not extremely elongate. Posterior apodeme of coxal field II not observed. Ventral median hysterosomal apodeme not observed. Coxal apodemes III-IV ending freely, not fused to each other medially. Coxal setae *1a* filiform. Coxal setae *4a* and *4b* not clearly observed. Attachment organ well developed, wider than long, positioned near posterior end of body, without median apodeme posterior to attachment organ and striated posterior membrane. Conoidal setae of attachment organ (*ps_1_* and *ps_2_*) arranged in a concave pattern; external conoidal setae (*ps_2_*) of attachment organ lateral to well-developed median sucker (*ad*_1+2_). Cuticular suckers of attachment organ not observed. Genital papillae not observed.

Legs. Genu I with 2 subequal solenidia (σ' and σ''). Genua III-IV subequal to tibiae III-IV. Dorsal solenidion (σ) and ventral seta *nG* not observed on genu III. Tibiae I-II similar in length to corresponding genua, with 1 ventral seta (*gT* I-II present, *hT* I-II absent). Tibial setae IV (*kT*) nearly as long as the length of genu-tarsus IV. Empodial claws simple, claw-like, not enlarged, hook-like or twisted, similar in size and form on tarsi III-IV. Tarsus I with solenidion ω_2,_ setae *aa* and *ba* present; solenidia ω_1_ and ω_2_ basal on tarsus, solenidion ω_3_ apical. Tarsi I-II with setae *aa* lateral and somewhat apical to solenidion ω_1_; apical setae *f* foliate, setae nearly filiform, without sucker-like apex ("saucer"). Tarsi I-IV normally developed, not attenuate, with foliate (regular, not very broad) and filiform setae, spine-like setae absent; tarsi III-IV not projecting at an angle from tibiae III-IV. Tarsi I-II only with setae *d* distinctly longer than respective tarsi; other tarsal setae at most as long as the tarsus length. Tarsi I-III distinctly longer than wide; tarsus IV slightly longer than wide. Tarsus III with 1 setae (*d)* longer than the length of legs III. Tarsus IV with 2 apical setae (*d* and *ba*) longer than the length of leg IV, 1 apical seta (*f*) as long as the length of genu-tarsus IV, and 1 seta (*r*) nearly as long as the length of genu-tarsus IV.

*Remarks*. This genus is based on a single species, *Levantoglyphus* *sidorchukae* n. sp. found in Cretaceous amber of Lebanon (see below). Unobserved character states are listed in the Remarks section under the family description above.

**†*Levantoglyphus* *sidorchukae* Klimov and Sidorchuk, n. sp.** (Figs 2–4, Supplementary Figs S1-8)

*Etymology*. The new species is named after the late Ekaterina (Katya) Sidorchuk who made major contribution in palaeoacarology. She initiated this project and accomplished critical tasks during its early stages (polishing the amber piece and partial imaging).

*Material*. Holotype: Heteromorphic deutonymph, specimen number 1213A (Azar collection), dorsoventral orientation, LEBANON: Mouhafazet Jabal Loubnan [Governorate of Mount Lebanon], Caza Baabda [Baabda District], Hammana - Mdeyrij outcrop, date and collector unknown (Natural History Museum of the Lebanese University, Faculty of Sciences II, Fanar, Lebanon).

Paratype: Heteromorphic deutonymph, specimen number 1213B (Azar collection), lateral orientation, same amber piece as holotype.

The single amber piece containing the holotype (1213A) and paratype (1213B) is housed in the Natural History Museum of the Lebanese University, Faculty of Sciences II, Fanar, Lebanon. The piece is mounted in epoxy resin between two coverslips.

Geological map of this locality is given in Azar, et al. ^2^. The age of the outcrop is Lower Barremian, ca. 129 My ^3,4^.

*Diagnosis*. As for the genus (see above).

*Description (heteromorphic deutonymph).* This species belongs to a monotypic genus having remote similarities with modern astigmatid mites. Because there is no other species to compare to (or even make an informed guess based on other astigmatid mites), we consider that the family and generic descriptions above are equivalent to the species description. Basic measurements are as follows: length of idiosoma 232(160), width 116 μm; propodosoma 96(65) μm long; hysterosoma 136(98) μm long (measurements are given for the holotype, and, in parentheses, for the paratype). Other measurements and species-level diagnostic character states can be derived from photographs (Figs 2–4, Supplementary Figs S1-8).

*Remarks*. Similarly to heteromorphic deutonymphs of crown group Astigmata, the fossil mite is well sclerotized, has the attachment organ and many foliate tarsal setae, and long apical setae on tarsi III-IV. Based on comparison with living mites, these character states collectively suggest that *Levantoglyphus* was phoretic on arthropods. But unlike all extant Astigmata, the gnathosoma, including the rutella and chelicerae (main structures participating in shredding food particles during feeding), are well developed in the fossil mite. These and some other character states, such as presence of setae *ba* on tarsi III-IV and setae *aa* on tarsi II, suggest that these mites have no close modern relatives, and therefore belong to the stem group Astigmata. We also note that the astigmatid genus *Schizoglyphus* (Schizoglyphidae) also has a number of plesiomorphic character states (3-segmented palps, 3 pairs of genital papillae) ^5^ suggesting its basal position, but in comparison with *Levantoglyphus* its gnathosoma is reduced (similarly to other Astigmata) and tarsal setae aa II and *ba* III-IV are absent. *Levantoglyphus* *sidorchukae* differs from an undescribed astigmatid deutonymph from the Cretaceous termite *Lebanotermes veltzae* ^6^ by the elongated propdosoma (much shorter in the termite mite).

**References**

1 Reuter, E. Zur Morphologie und Ontogenie der Acariden mit besonderer Berücksichtigung von *Pediculopsis graminum* (E. Reut.). *Acta Societatis Scientiarum Fennicae* **36**, 1-288 (1909).

2 Azar, D., Gèze, R. & Acra, F. in *Biodiversity of fossils in amber from the major world deposits.* (ed David Penney) 271-298 (Siri Scientific Press, 2010).

3 Granier, B., Toland, C., Gèze, R., Azar, D. & Maksoud, S. Some steps toward a new story for the Jurassic - Cretaceous transition in Mount Lebanon. *Carnets De Geologie* **16**, 247-269 (2016).

4 Maksoud, S., Azar, D., Granier, B. & Gèze, R. New data on the age of the Lower Cretaceous amber outcrops of Lebanon. *Palaeoworld* **26**, 331-338, doi:10.1016/j.palwor.2016.03.003 (2017).

5 OConnor, B. M. in *A Manual of Acarology. Third Edition.* (eds G. W. Krantz & D. E. Walter) 565-657 (Texas Tech University Press, 2009).

6 Engel, M. S. *et al.* New, primitive termites (Isoptera) from Early Cretaceous ambers of France and Lebanon. *Palaeodiversity* **4**, 39-49 (2011).


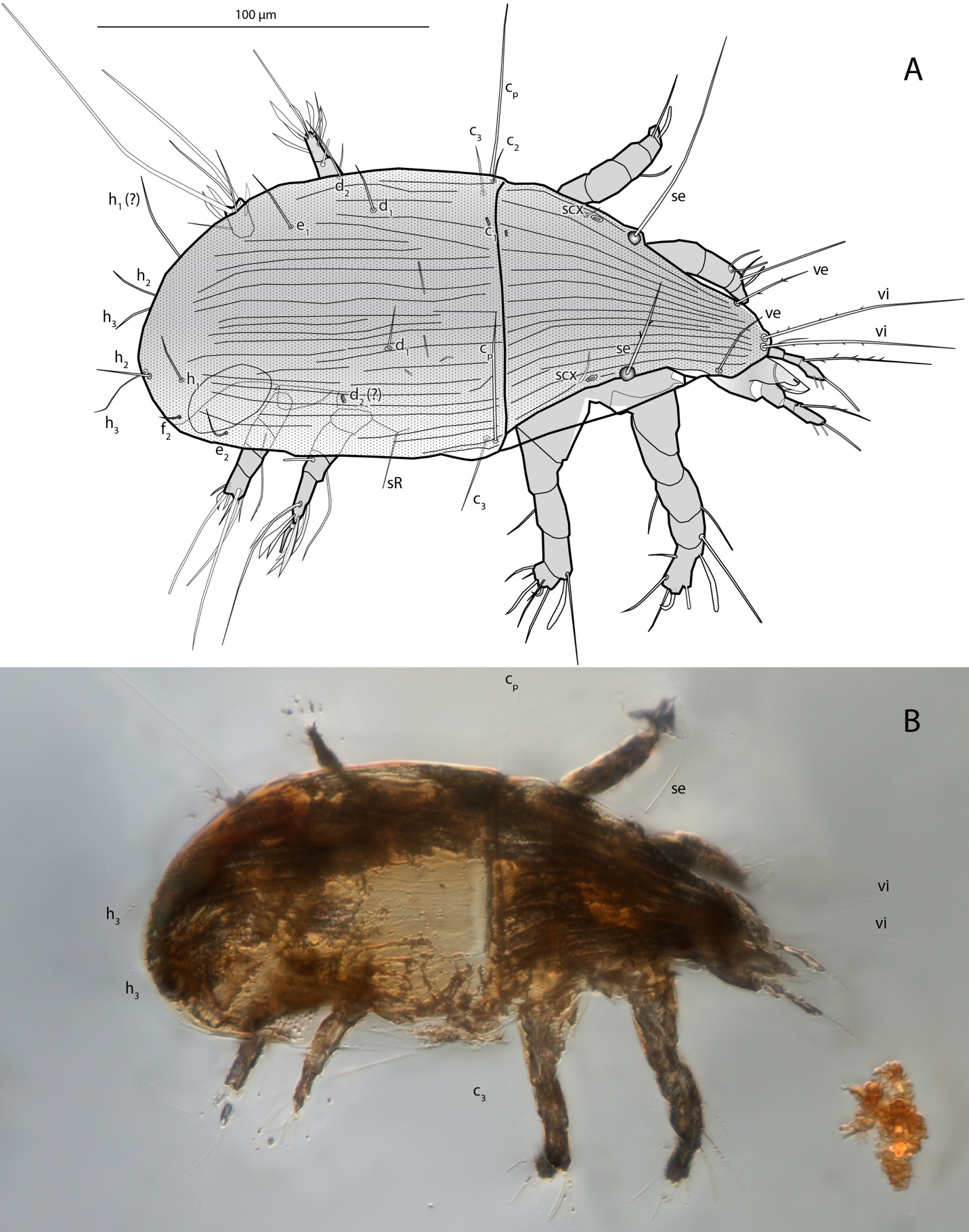
**Figure** **S1**. *Levantoglyphus* *sidorchukae* n. gen. and sp., phoretic heteromorphic deutonymph (paratype 1213B), dorsolateral view. **A**, line art. **B**, DIC photo (only well visible structures are labelled).
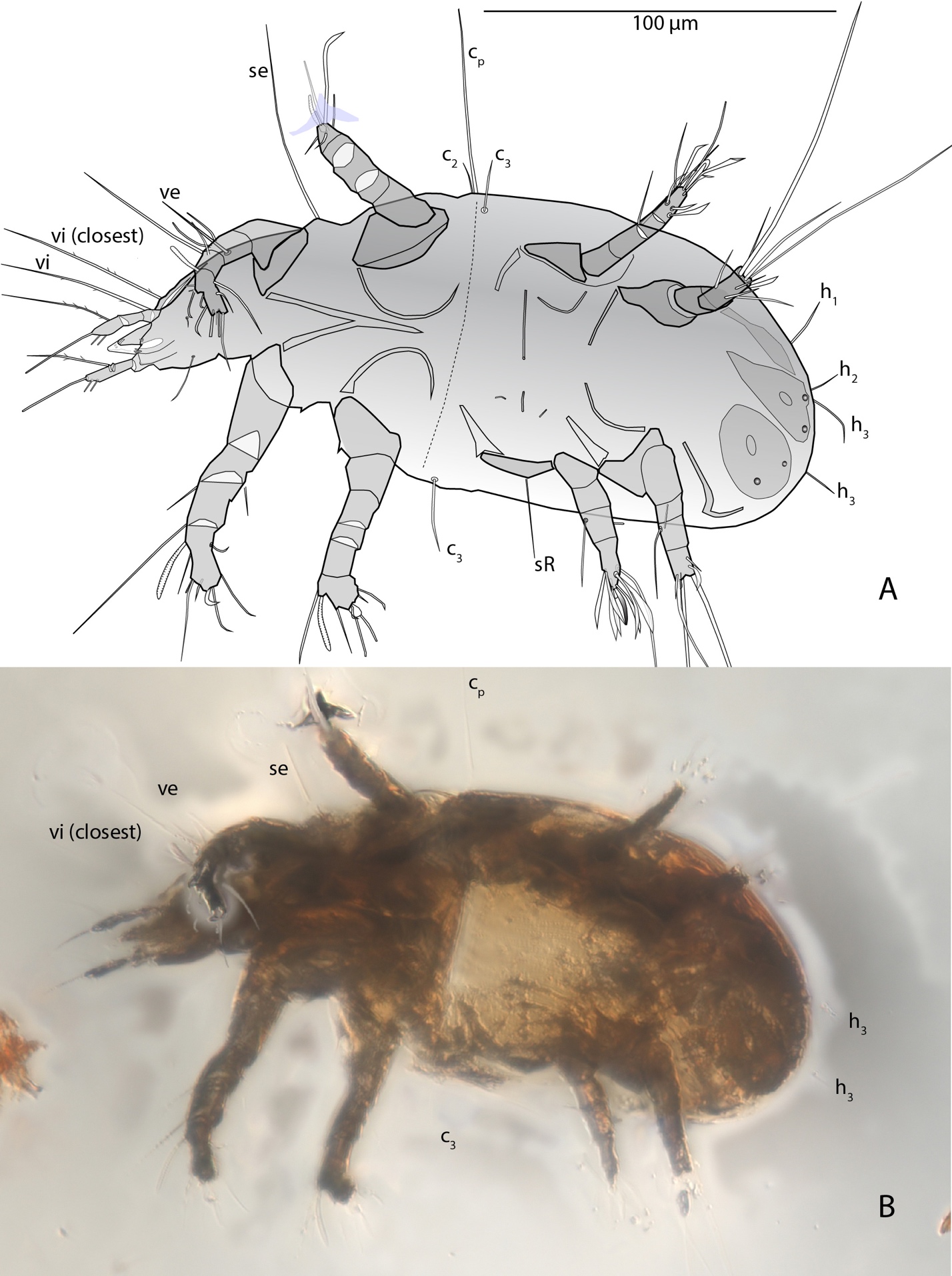


**Figure** **S2**. *Levantoglyphus* *sidorchukae* n. gen. and sp., phoretic heteromorphic deutonymph (paratype 1213B), ventrolateral view. **A**, line art. **B**, DIC photo (only well visible structures are labelled).

**
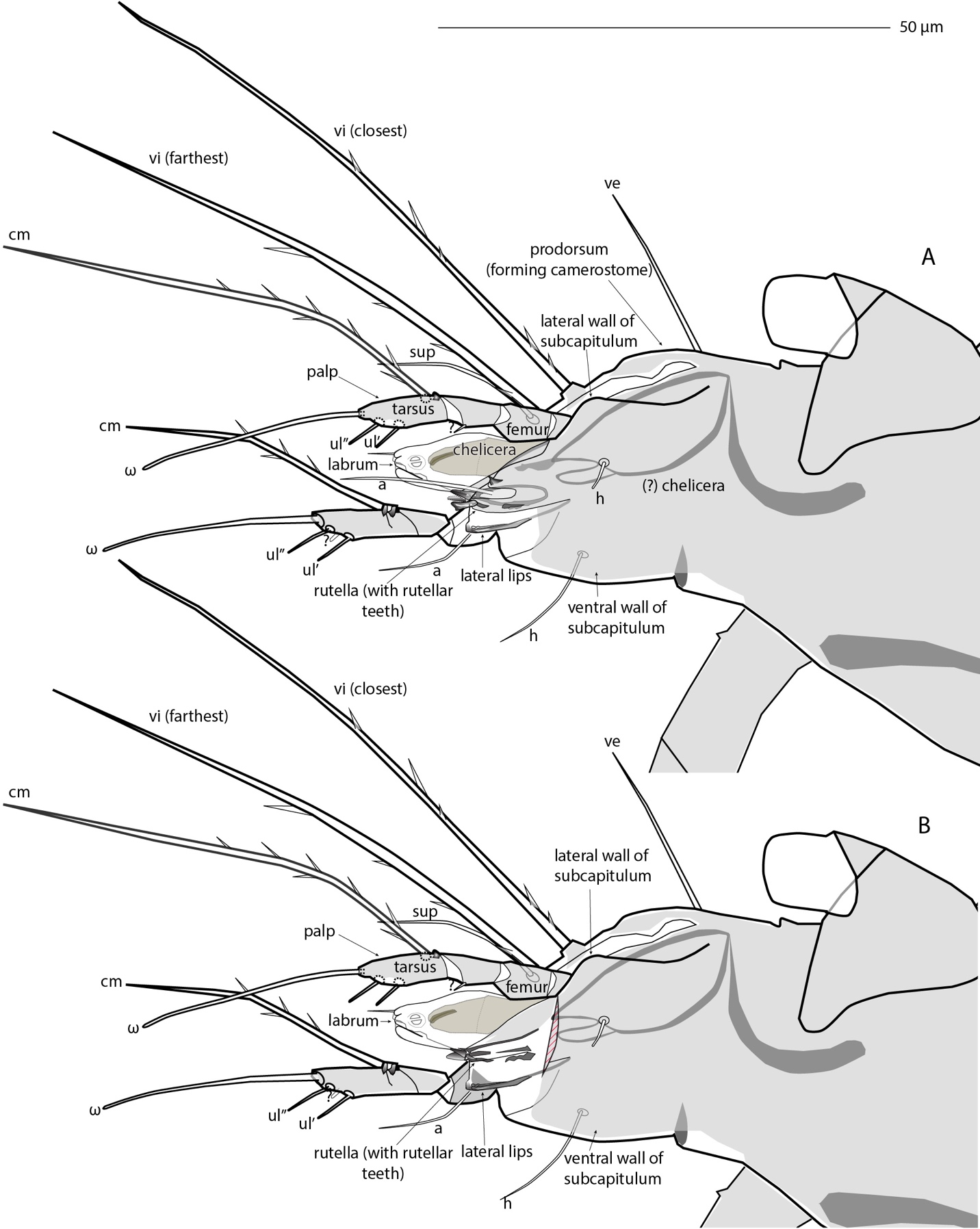
Figure** **S3**. *Levantoglyphus* *sidorchukae* n. gen. and sp., phoretic heteromorphic deutonymph (paratype 1213B), gnathosoma, line art. **A**, ventrolateral view. **B**, same but anterolateral portion of subcapitulum removed (red striation) to show median structures.

**
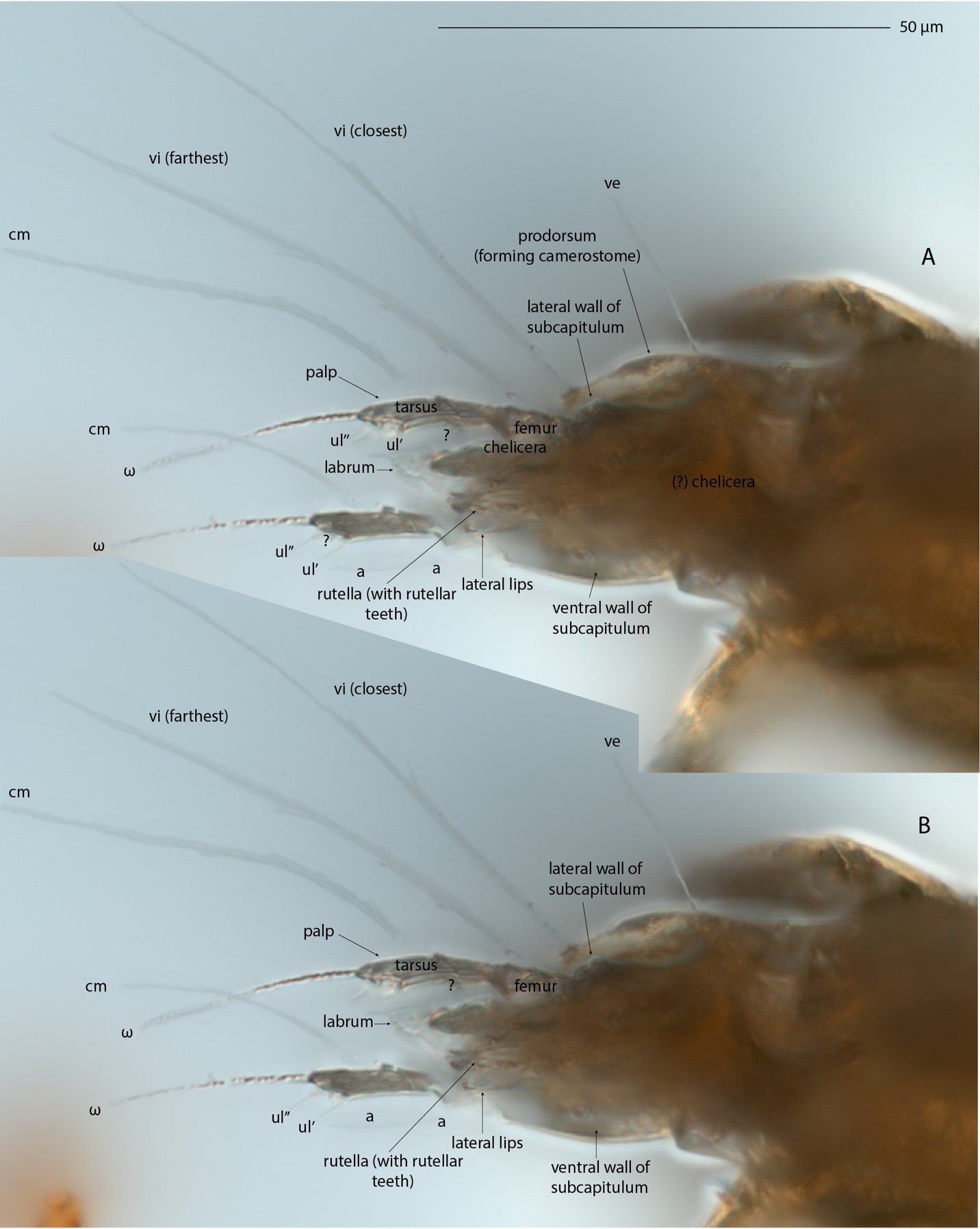
Figure** **S4**. *Levantoglyphus* *sidorchukae* n. gen. and sp., phoretic heteromorphic deutonymph (paratype 1213B), gnathosoma, DIC photos. **A**, ventrolateral view. **B**, same but anterolateral portion of subcapitulum removed to show median structures.

**
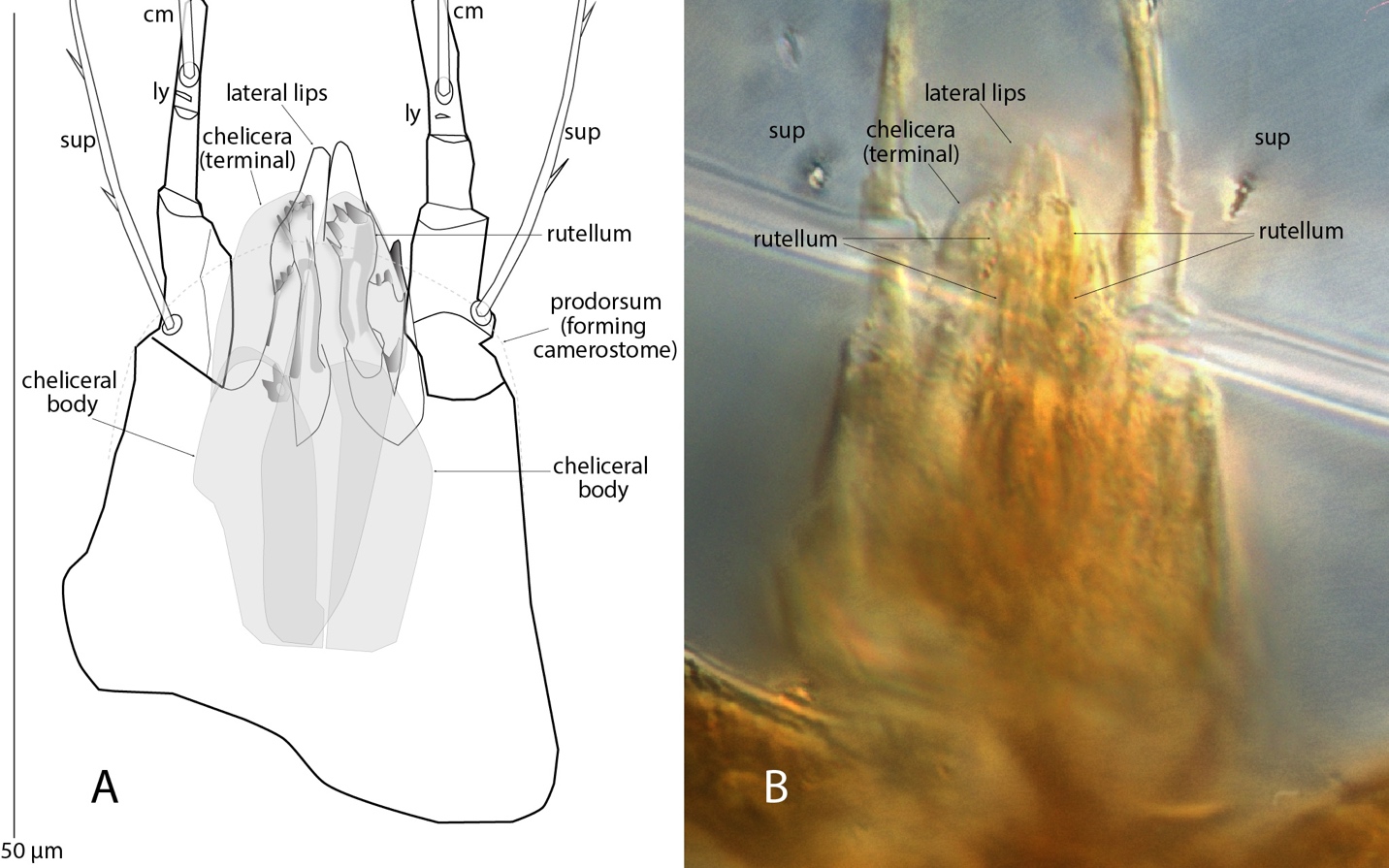
Figure** **S5**. *Levantoglyphus* *sidorchukae* n. gen. and sp., phoretic heteromorphic deutonymph (holotype 1213A), gnathosoma, midventral optical section at level of rutellar teeth. **A**, line art. **B**, DIC photo (low contrast structures not labelled).


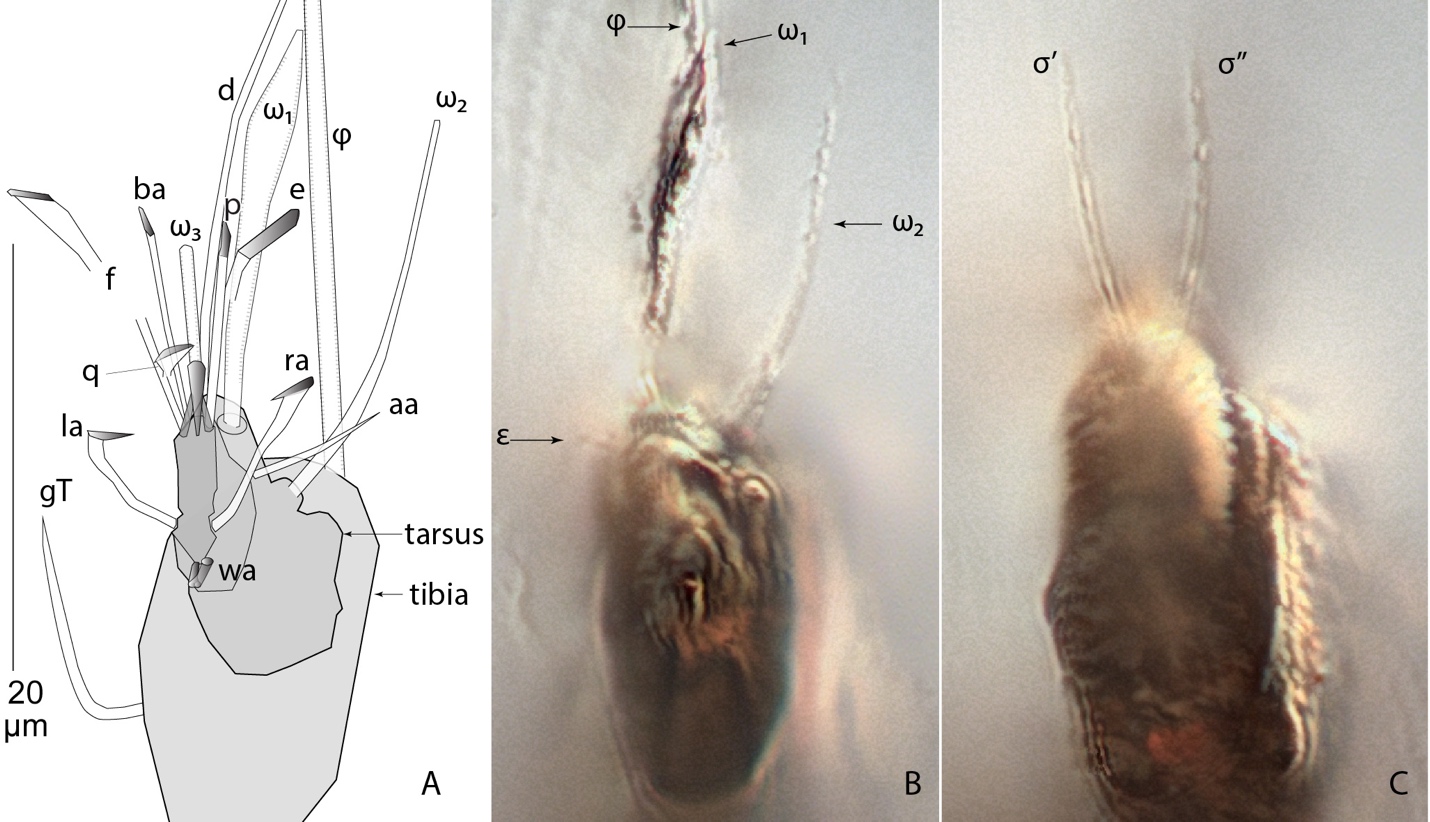


**Figure S6**. *Levantoglyphus* *sidorchukae* n. gen. and sp., phoretic heteromorphic deutonymph (holotype 1213A), left leg I, axial view. **A**, line art. **B**, cross-section at level of tarsal solenidia ω_1_, ω_2_ and famulus (ε), DIC photo. **C**, cross-section at level of genual solenidia (σ' and σ''), DIC photo.

**
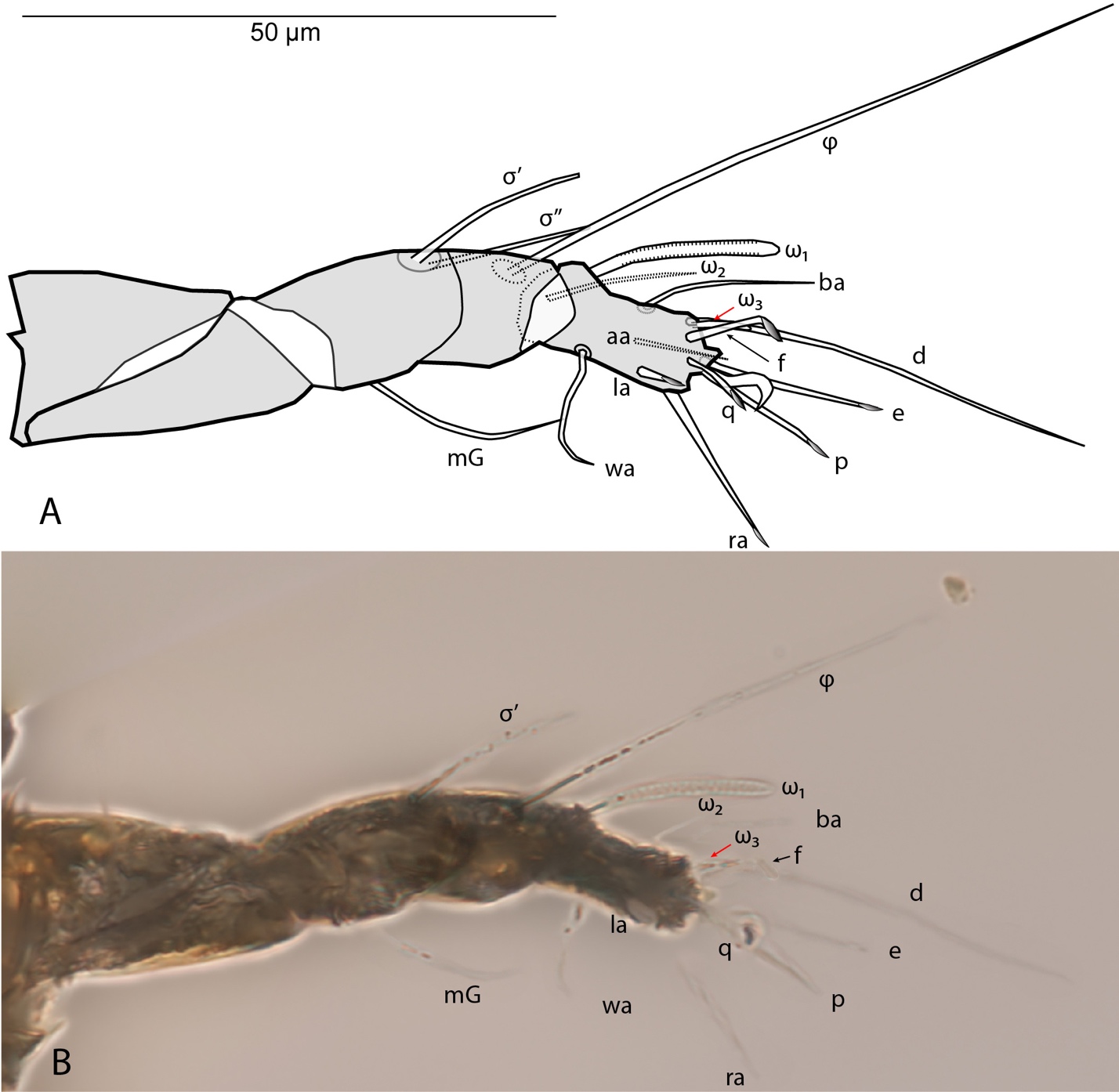
Figure** **S7**. *Levantoglyphus* *sidorchukae* n. gen. and sp., phoretic heteromorphic deutonymph (paratype 1213B), right leg I, ventrolateral view (most of anterior side shown). **A**, line art. **B**, DIC photo.

**Figure** **S8**. *Levantoglyphus* *sidorchukae* n. gen. and sp., phoretic heteromorphic deutonymph (holotype 1213A), left tarsus-tibia II, axial view, line art.
